# Autophagy couteracts weight gain, lipotoxicity and pancreatic *β*-cell death upon hypercaloric pro-diabetic regimens

**DOI:** 10.1038/cddis.2017.373

**Published:** 2017-08-03

**Authors:** Álvaro F Fernández, Clea Bárcena, Gemma G Martínez-García, Isaac Tamargo-Gómez, María F Suárez, Federico Pietrocola, Francesca Castoldi, Lorena Esteban, Elena Sierra-Filardi, Patricia Boya, Carlos López-Otín, Guido Kroemer, Guillermo Mariño

**Affiliations:** 1 Instituto de Investigación Sanitaria del Principado de Asturias, Oviedo, Spain; 2 Departamento de Bioquímica y Biología Molecular, Instituto Universitario de Oncología, Universidad de Oviedo, Oviedo, Spain; 3 Departamento de Biología Fundamental, Universidad de Oviedo, Oviedo, Spain; 4 Equipe 11 labellisée par la Ligue Nationale Contre le Cancer, INSERM U1138, Centre de Recherche des Cordeliers, Paris, France; 5 Metabolomics and Molecular Cell Biology Platforms, Gustave Roussy, Villejuif, France; 6 Université Paris Descartes, Sorbonne Paris Cité, Paris, France; 7 Sotio a.c., Prague, Czech Republic; 8 Departament of Cellular and Molecular Biology, Centro de Investigaciones Biológicas, CSIC, Madrid, Spain; 9 Pôle de Biologie, Hôpital Européen Georges Pompidou, AP-HP, Paris, France

## Abstract

In the last years, autophagy has been revealed as an essential pathway for multiple biological processes and physiological functions. As a catabolic route, autophagy regulation by nutrient availability has been evolutionarily conserved from yeast to mammals. On one hand, autophagy induction by starvation is associated with a significant loss in body weight in mice. Here, we demonstrate that both genetic and pharmacological inhibition of the autophagy process compromise weight loss induced by starvation. Moreover, autophagic potential also impacts on weight gain induced by distinct hypercaloric regimens. *Atg4b*-deficient mice, which show limited autophagic competence, exhibit a major increase in body weight in response to distinct obesity-associated metabolic challenges. This response is characterized by the presence of larger adipocytes in visceral fat tissue, increased hepatic steatosis, as well as reduced glucose tolerance and attenuated insulin responses. Similarly, autophagy-deficient mice are more vulnerable to experimentally induced type-I diabetes, showing an increased susceptibility to acute streptozotocin administration. Notably, pharmacological stimulation of autophagy in wild-type mice by spermidine reduced both weight gain and obesity-associated alterations upon hypercaloric regimens. Altogether, these results indicate that systemic autophagic activity influences the resilience of the organism to weight gain induced by high-calorie diets, as well as to the obesity-associated features of both type-1 and type-2 diabetes.

Autophagy is well known to control the mass of individual cells because it constitutes the most efficient pathway for the degradation of cytosolic proteins and protein aggregates,^1^ as well as the sole pathway for the digestion and recycling of cytoplasmic organelles.^2^ Thus, in response to nutrient starvation or different external stimuli, cells reduce their biomass in an autophagy-dependent fashion,^3, 4, 5^ accessorily degrading macromolecules into the building blocks of adaptive responses, as well as into substrates of energy-producing reactions.^6^

This catabolic process is one of the prime responses to nutrient depletion at the whole-body level. In response to starvation, mice manifest a major reduction in body weight (~20% in 48 h) as they mount a major autophagic response that is detectable in most if not all organs of the body.^7^ In this context, it is well known that autophagy is required for the healthspan- and lifespan-extending effects of caloric restriction, at least in model organisms.^8^ Indeed, the knockout of genes indispensable for autophagy is compatible with life in yeast, nematodes or flies, although the resulting autophagy deficiency reduces the overall fitness of these organisms, compromises their longevity and abolishes the positive effects of caloric restriction on the aging process and life expectancy.^8^ In mice, the loss of essential autophagy genes either results into neonatal death shortly after birth^9, 10, 11^ or causes embryonic lethality due to developmental defects.^12, 13, 14, 15^ This implies that the impact of autophagy on caloric restriction has not been fully addressed in mammals, due to the lack of appropriate animal models.

Several mammalian autophagy genes, as *Atg3*, *Atg5*, *Atg7, Ambra1, FIP200* and *Becn1*, among others, operate in a non-redundant way in central hubs of the autophagic pathway, meaning that their knockout causes a complete (and lethal) defect in autophagy.^16^ In contrast to the former cases, mammals possess several orthologs of yeast Atg4 protease that together form the Atg4 protein family, composed by ATG4A, ATG4B, ATG4C and ATG4D.^17, 18^ Hence, the knockout of one single member of the family, for instance *Atg4b,* is compatible with a close-to-normal development before and beyond birth.^19, 20^ Nonetheless, *atg4b*^−/−^ mice exhibit a significant reduction of their autophagic potential in multiple distinct organs. Thus, autophagic flux, as well as the depletion of the autophagic substrate SQSTM1/p62, are diminished in both fed and starved *atg4b*^−/−^ mice compared with wild-type (WT) littermates.^19^

Recently, we observed that induction of autophagy by a novel caloric restriction mimetic, hydroxycitrate, induces weight loss in control WT mice, yet failed to do so in *atg4b*^−/−^ animals.^21, 22^ These findings established a precedent indicating that autophagy might control body mass in conditions as starvation and prompted us to investigate the impact of systemic autophagy reduction on the control of weight gain induced by high-calorie regimens. Here, we report that both genetic and chemical attenuation of autophagic flux account for a reduction in starvation-induced weight loss. Moreover, autophagy deficiency at the whole-body level predisposes to obesity, hyperinsulinemia and insulin intolerance upon diverse metabolic challenges, and also increases the severity of experimentally induced type-I diabetes. Finally, we show that the detrimental effects of hypercaloric diets in WT mice can be mitigated by pharmacological induction of autophagy *in vivo*, delineating new promising therapeutic strategies against obesity-related pathologies.

## Results

### Reduced starvation-induced weight loss in autophagy-deficient mice

**Figure 1:**
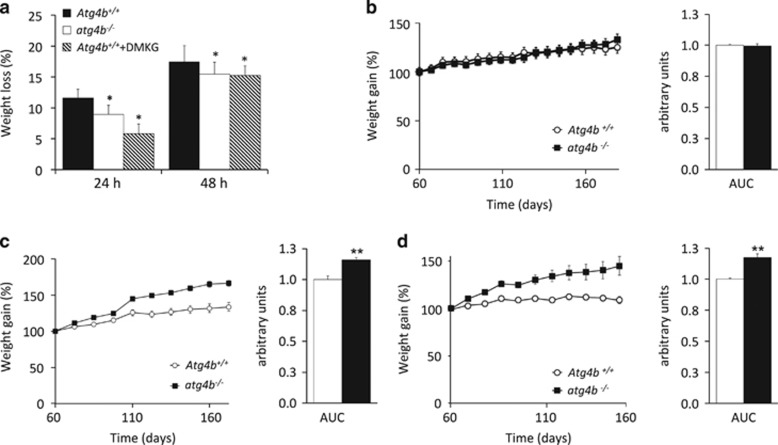
Effects of systemic autophagy deficiency on weight changes induced by starvation and hypercaloric dietary regimens. (**a**) *atg4b*^−/−^ Mice show reduced weight loss (depicted as the percentage of the initial weight) upon either 24- or 48-h of starvation, as compared with age-matched WT littermates. This was also observed in control mice treated with the autophagy inhibitor dimethyl-*α*-ketoglutarate (DMKG). (**b**) Weight curves of WT or *atg4b*^−/−^ mice fed *ad libitum* with standard rodent diet without any supplementation in drinking water. Bar graph shows area under the curves (AUC). (**c**) Weight curves of WT or *atg4b*^−/−^ mice fed *ad libitum* with a high-fat diet containing 42% fat and without any supplementation in drinking water. Bar graph shows AUC. (**d**) Weight curves of WT and *atg4b*^−/−^ mice fed *ad libitum* with standard rodent diet with 30% sucrose supplementation in drinking water. Bar graph shows AUC. For starvation-related experiments in mice, at least 6 animals per genotype/condition were used. For specific diets experiments in mice, at least 12 animals per genotype/condition were used. *P-value <0.05 and **P-value <0.01 in two-tailed student’s *t*-test for each of the depicted points

*atg4b*^−/−^ Mice generated by crossing heterozygous *Atg4b*^+/-^ parents are born at the expected Mendelian frequency and appear phenotypically normal, with the sole exception that 15–20% of the mice exhibit perturbed equilibrioception due to deficient otoconial biogenesis.^19^ In response to starvation for 24–48 h, such mice also exhibit a clear defect in the induction of autophagy in multiple distinct organs.^19^ This autophagy defect of *atg4b*^−/−^ mice was associated to a reduced weight loss upon fasting when compared with WT littermates (Figure 1a). Similarly, suppression of starvation-induced autophagy by injection of the autophagy inhibitor *α*-ketoglutarate precursor, dimethyl *α*-ketoglutarate,^21^ blunted weight loss in WT mice (Figure 1a). Hence, different methods to inhibit starvation-induced autophagy (deficiency of *Atg4b*, as well as pharmacological inhibition with the anaplerotic substrate α-ketoglutarate) have a similar effect on weight reduction.

### Excessive weight gain of autophagy-deficient mice fed with hypercaloric diets

In order to further analyze a possible role for systemic autophagy in weight regulation, we next decided to test whether the partial but systemic autophagy impairment of *atg4b*^−/−^ mice could affect body weight gain under hypercaloric diets. As shown by area under the curve (AUC) comparison, adult (60 day-old) *atg4b*^−/−^ mice and littermate WT controls exhibited a similar age-associated weight gain over the subsequent 120 days if fed standard chow *ad libitum* (Figure 1b). In contrast, if *atg4b*^−/−^ mice were fed a Western style high-fat diet, they exhibited a statistically significant increase in weight gain, as compared with WT animals (Figure 1c). Similarly, there was also a significant difference in the response of *atg4b*^−/−^ and WT mice to continuous supplementation of the drinking water with 30% sucrose, a model of experimentally induced metabolic syndrome. In WT mice, this regimen fails to induce a major weight gain, yet causes hepatic steatosis and insulin resistance.^23, 24^ Unexpectedly, but in accord with our previous results, *atg4b*^−/−^ mice exhibited a steady weight gain that reached ~50% after 100 days of sucrose treatment, which was significantly higher that what was observed in WT controls under this same regimen (Figure 1d).

**Figure 2:**
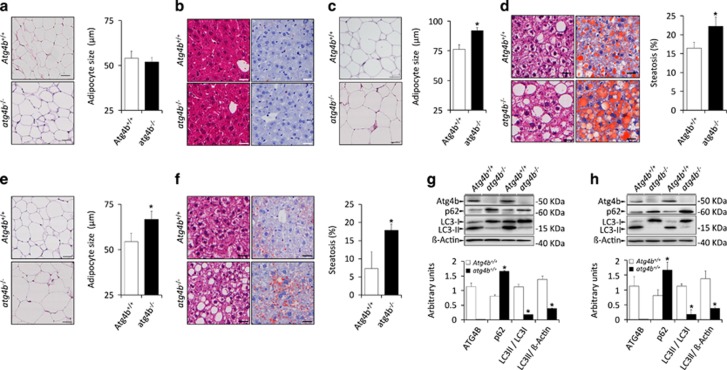
Obesity-related features of *atg4b*^−/−^ mice fed with hypercaloric regimens. (**a**) Left, representative pictures of adipocytes from H&E sections of white adipose tissue in untreated WT and a*tg4b*^−/−^ mice. Right, quantification of the data. (**b**) Left, representative pictures from H&E sections (left) and Oil Red staining (right) of livers from untreated WT and *atg4b*^−/−^ mice showing the absence of hepatic steatosis. (**c**) Left, representative pictures of adipocytes from H&E sections of white adipose tissue in WT and *atg4b*^−/−^ mice upon high-fat diet. Right, quantification of the data. (**d**) Left, representative pictures from H&E sections (left) and Oil Red staining (right) of livers from WT and *atg4b*^−/−^ mice upon high-fat diet showing an increase of hepatic steatosis in *atg4b*^−/−^ mice. Right, quantification of the data. (**e,f**) Representation of equivalent data to those shown in **c** and **d** for sucrose-treated WT and *atg4b*^−/−^ mice. (**g,h**) Immunoblotting analyses of ATG4B, SQSTM1/p62 and LC3B in white adipose (**g**) and liver (**h**) tissues from mice subjected for 60 days to high-fat diet, showing the absence of ATG4B protein, the reduction of autophagosome-associated LC3B-II and the accumulation of the specific autophagic substrate SQSTM1/p62, all indicative of reduced autophagic flux. Graph bars show quantification of the data depicted in Immunoblotting panels. *P-value <0.05 in two-tailed student’s *t*-test. Scale bars, 30 *μ*m

Although *atg4b*^−/−^ and WT mice were undistinguishable with respect to the mass and size of adipocytes (Figure 2a), *atg4b*^−/−^ mice that received a high-fat diet or sweetened water exhibited a more abundant subcutaneous and visceral fat than WT controls, with an abnormal increase in the mean diameter of adipocytes (Figures 2c and e). Moreover, *atg4b*^−/−^ mice only showed increased hepatic steatosis with respect to controls when challenged with high-fat diet or sugar-enriched water (Figures 2d and f), as these differences were not present when mice were fed standard diet (Figure 2b). This suggests that the observed increase of fat accumulation is not limited to white adipose tissue, but is rather a consequence of a systemic obesity-linked metabolic alteration. Interestingly, circulating values of alanine and aspartate aminotransferases in *atg4b*^−/−^ mice were comparable to those from WT mice in all different experimental settings (Supplementary Figures 1A and B). This indicates that *Atg4b* deletion itself does not lead to liver damage, contrasting with previous studies in which liver autophagy is totally impaired.^25, 26^ Moreover, plasma values of certain pro-inflammatory cytokines and chemokines, including IFN*γ* or IL-6, were slightly augmented in *atg4b*^−/−^ mice when subjected to high-calorie regimens, in accord with previous studies linking higher pro-inflammatory responses the absence of ATG4B,^20^ although these differences failed to reach statistical significance (Supplementary Figure 1C). Irrespectively of the aforementioned observations regarding circulating parameters, WT and *atg4b*^−/−^ mice showed similar degrees of inflammatory infiltration both in liver and adipose tissue either untreated or upon high-calorie regimens (Supplementary Figures 1D–I). Thus, increased meta-inflammation is likely not a major cause for the higher obesity observed in autophagy-deficient mice, which may be mainly linked to the reduced autophagic flux that *atg4b*^−/−^ mice manifest in all tissues,^19^ including liver and white fat (Figures 2g and h).

### Obesity-related biochemical features in autophagy-deficient mice

**Figure 3:**
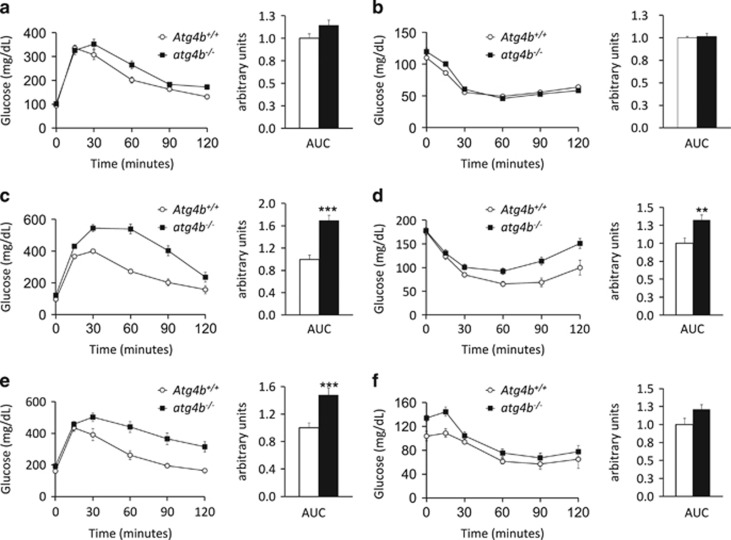
Glucose- and insulin-tolerance in *atg4b*^−/−^ mice fed with hypercaloric regimens. (**a**) Intraperitoneal glucose tolerance test (IPGTT), and (**b**) intraperitoneal insulin tolerance test (IPITT), in untreated WT and *atg4b*^−/−^ mice (*n*=6). (**c,d**) Intraperitoneal glucose test tolerance (IPGTT), and intraperitoneal insulin tolerance test (IPITT), in WT and *atg4b*^−/−^ mice upon high-fat diet (*n*=6). (**e**,**f**) Equivalent analyses in sucrose-treated WT and *atg4b*^−/−^ mice (*n*=6). Bar graph shows area under the curves (AUC). Error bars represent means±S.E.M. *P-value<0.05

**Figure 4:**
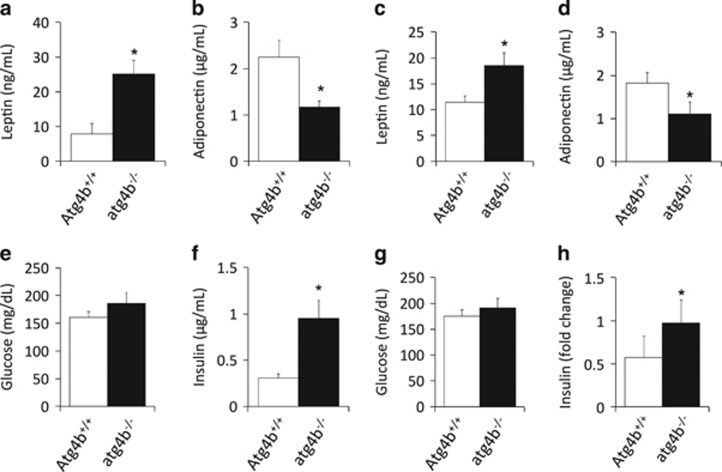
Analysis of obesity-linked biochemical parameters in *atg4b*^−/−^ mice. (**a-d**) Circulating levels of leptin (**a** and **c**) and adiponectin (**b** and **d**) in high-fat fed (**a** and **b**) and sucrose-treated *atg4b*^−/−^ mice (**c** and **d**), as compared with their corresponding age-matched WT controls (*n*=6). (**e–h**) Blood glucose levels (**e** and **g**) and plasma insulin levels (**f** and **h**) in in high-fat fed (**e** and **f**) and sucrose-treated WT and *atg4b*^−/−^ mice (**g** and **h**). Bars represent means±S.D. (*n*=6). *P-value<0.05

Intrigued by the aforementioned observations, we determined obesity-associated biochemical features in either unchallenged or treated *atg4b*^−/−^ mice and their WT controls. As shown by AUC comparison, both glucose tolerance test (GTT) and insulin tolerance test (ITT) showed no significant differences between untreated WT and autophagy-deficient mice (Figures 3a and b). However, upon challenge with high-fat diet or sucrose-enriched water, mutant mice maintained significantly higher glucose levels for a longer period than their corresponding WT controls during the GTT (Figures 3c and e). Moreover, when fed with hypercaloric regimens, the decrease in glucose level induced by insulin injection was significantly attenuated in *atg4b*^−/−^ animals compared with WT controls (Figures 3d and f). When unchallenged, *atg4b*^−/−^ mice show normal circulating glucose, insulin, leptin or adiponectin levels (data not shown). In contrast, upon high-fat chow feeding or sucrose treatment, autophagy-incompetent mice exhibit significant differences in many of these parameters. Specifically, leptin serum levels were higher and adiponectin concentrations were lower in *atg4b*^−/−^ mice than in WT controls (Figures 4a–d). Although blood glucose levels were slightly higher in mutant mice upon hypercaloric diets, the differences were not statistically significant (Figures 4e and g). In contrast, circulating insulin concentrations were substantially higher in *atg4b*^−/−^ mice subjected to high-calorie regimens, as compared with WT controls, which may account for their reduced insulin sensitivity (Figures 4f and h). The surge in circulating insulin levels was accompanied by an increase in the size and number of pancreatic islets, as well as an increase in relative *β*-cell mass in *atg4b*^−/−^ mice subjected to high-calorie diets. Interestingly, this increase in the number and size of *β*-cells patches was not detected in the corresponding age-matched WT littermates (Supplementary Figure 2).

### Reduced metabolism of autophagy-deficient mice fed with hypercaloric diets

**Figure 5:**
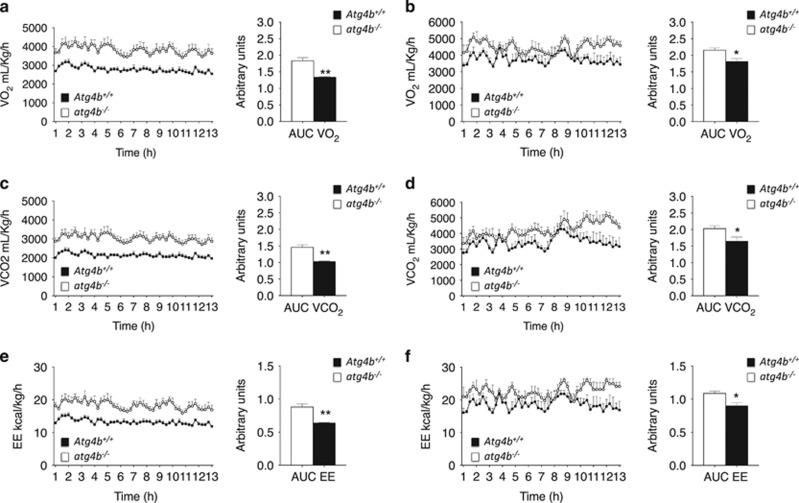
Analysis of respiration and energy expenditure in *atg4b*^−/−^ mice fed upon high-calorie regimens. (**a**) VO_2_ consumption (ml/kg/hour) during dark phase (12 h) (left) and area under the curve of the VO_2_ consumption (right) for WT and *Atg4b*-null mice upon high-fat diet (*n*=4). (**b**) Same representation as in **a** for sucrose-treated WT and *Atg4b*-null mice. (**c,d**) VCO_2_ production for WT and *Atg4b*-null mice fed with high-fat diet (**c**), or sucrose treated (**d**) (*n*=4). (**e,f**) Energy expenditure (Kcal/kg/hour) during dark phase (12 h) and area under the curve of the energy expenditure (right) for *WT* and *Atg4b*-null mice fed with high-fat diet (**e**), or sucrose treated (**f**) (*n*=4). Data represent mean±S.E.M. *P<0.05, **P<0.01

To understand the cause of the obesity-linked alterations observed in *Atg4b*-deficient mice, we examined daily food intake and energy expenditure by means of the Oxymax/CLAMS system. Interestingly, the high-fat or sugar-induced obesity of *atg4b*^−/−^ mice could neither be attributed to increased caloric intake nor to a significantly reduced locomotor activity. Surprisingly, *atg4b*^−/−^ mice tended to reduce their food and water intake under high-fat diet or sucrose treatment, although this trend did not reach statistical significance (Supplementary Figure 3). However, *atg4b*^−/−^ mice did show both lower oxygen consumption (VO_2_), and lower carbon dioxide release (VCO_2_), when subjected to high-calorie regimens (Figures 5a–d), which was not observed under standard diet (Supplementary Figures 4A and B). This led to a decrease of energy expenditure in *atg4b*^−/−^ mice, as compared with WT animals, when subjected to both high-calorie regimens (Figures 5e and f). However, no difference in energy expenditure was observed in *ad libitum* fed *atg4b*^−/−^ and WT mice receiving the standard chow (Supplementary Figure 4C). This alteration of the metabolic rate occurred without changes in the respiratory exchange ratio (RER), which reflects the relative use of carbohydrates *versus* lipids as a source of energy (Supplementary Figures 4D–F). These results suggest that reduced energy expenditure could be contributing to the observed increase of weight gain in autophagy-deficient mice.

Together, our results indicate that the partial and systemic reduction of autophagic activity of *atg4b*^−/−^ mice predisposes to high-fat and sucrose-induced obesity upon a similar, or even lower, calorie intake, by reducing energy expenditure, oxygen consumption and carbon dioxide release.

### Autophagy-deficient mice are more susceptible to experimentally induced type-I diabetes

**Figure 6:**
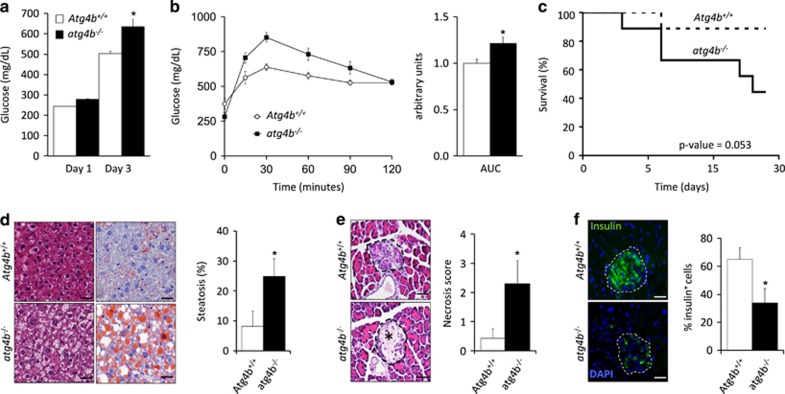
*atg4b*^−/−^ mice are more prone to streptozotocin-induced type-I diabetes. (**a**) Blood glucose levels of streptozotocin (STZ)-treated WT and *atg4b*^−/−^ mice at day 1 and day 3 after STZ injection (*n*=8). (**b**) Intraperitoneal glucose tolerance test (IPGTT) in STZ-treated WT and *atg4b*^−/−^ mice at day 3. Bar graph shows areas under curve (AUC) (*n*=8). (**c**) Kaplan–Meier graph of STZ-treated WT and *atg4b*^−/−^ mice showing a significant increment of mortality in the absence of ATG4B. (*n*=8). (**d**) Left, representative pictures from H&E sections (left panels) and Oil Red staining (right panels) of livers from STZ-treated WT and *atg4b*^−/−^ mice at day 3. Right, quantification of the data. Scale bars, 30 *μ*m. (**e**) Representative pictures from H&E sections of pancreatic *β*-cell islets at day 3 after STZ treatment (left). *β*-cell islets are surrounded by dashed lines. Necrotic area is marked by an asterisk. Right, quantification of necrotic stage in these samples. Scale bars, 60 *μ*m. (**f**) Immunofluorescence analysis against insulin showing specific loss of *β*-cell in *atg4b*^−/−^ at day 3 after STZ treatment (left). *β*-cell islets are surrounded by dashed lines. Right, quantification of the data. Data represent mean±S.E.M. *P-value<0.05. Scale bars, 60 *μ*m

In order to test whether the partial and systemic autophagy impairment of *Atg4b*-null mice might also predispose to experimentally induced type-I diabetes, we next decided to treat WT and autophagy-deficient mice with streptozotocin (STZ), Thus, mice were administered a single dose of STZ and after 3 days, blood glucose levels and glucose tolerance were evaluated. In concordance with our previous results, *Atg4b*-null mice showed a significant increase in blood glucose levels, as compared with the corresponding controls, as well as a reduced performance in the GTT (Figures 6a and b). These alterations were progressively exacerbated in autophagy-deficient mice and finally resulted in an increased mortality (60%), as compared with the corresponding controls (10%) (Figure 6c). Interestingly, STZ-treated *atg4b*^−/−^ mice showed clear signs of hepatosteatosis, which was hardly observed in equally treated WT mice (Figure 6d). Moreover, histological analyses of pancreas from STZ-treated mice revealed that loss of *β*-cell islets integrity was more pronounced in mutant mice, which showed increased signs of necrotic cell death and a reduced presence of healthy insulin-producing *β*-cells (Figures 6e and f). Altogether, these data show that the partial and systemic autophagy defect of *Atg4b*-null mice not only predisposes to diet-induced obesity, but also renders them more prone to pancreatic *β*-cell damage, also predisposing them to environmentally induced type-I diabetes.

### Induction of autophagy by spermidine prevents weight gain induced by high-fat diet

**Figure 7:**
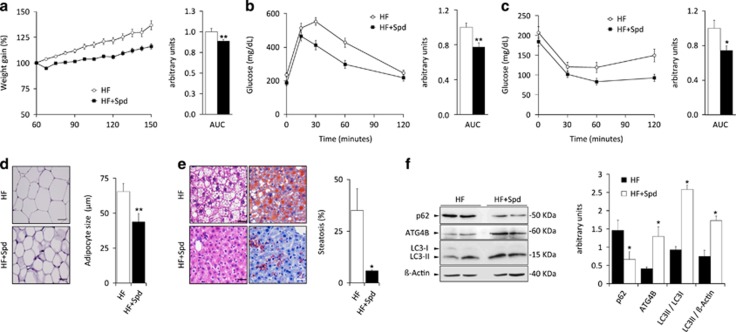
Autophagy induction by spermidine protects from the detrimental effects of high-fat diet in wild-type mice. (**a**) Weight curves of untreated and spermidine-treated WT mice (spd) fed with high-fat diet (HF) without any supplementation in drinking water. Bar graph shows area under the curves (AUC) (*n*=10). (**b**) Intraperitoneal glucose tolerance test (IPGTT), and (**c**) intraperitoneal insulin tolerance test (IPITT), in untreated and spermidine-treated high-fat fed WT mice. Bar graph shows area under the curves (AUC). (**d**) Left, representative pictures of adipocytes from H&E sections of white adipose tissue in untreated and spermidine-treated high-fat fed WT mice. Right, quantification of the data. (**e**) Left, representative pictures from H&E sections (left panels) and Oil Red staining (right panels) of livers from untreated and spermidine-treated high-fat fed WT mice. Right, quantification of the data. (**f**) Left, immuno-blotting analyses of ATG4B, LC3B and SQSTM1/p62 proteins in white adipose tissue extracts from either vehicle-treated or spermidine-treated WT mice subjected to high-fat diet, showing the effective increase of both ATG4B expression and autophagic flux in spermidine-treated mice. Right, quantification of the data. Data represent mean±SEM. Left, graph bars showing quantification of the data depicted in immuno-blotting panels. *P-value<0.05. Scale bars, 30 *μ*m.

Prompted by our results pointing to a protective role for stress-induced autophagy against different types of metabolic disturbances, we decided to evaluate whether therapeutic induction of autophagy would counteract the detrimental effects of pro-obesity regimens, including weight gain and its associated metabolic alterations observed upon hypercaloric challenge. To this aim, WT mice fed with Western style high-fat diet were given a daily dose of spermidine, a polyamine which induces autophagy *in vivo*.^22, 27^ Interestingly, spermidine-treated mice showed a significant reduction of weight gain, as compared with the corresponding high-fat diet fed controls (Figure 7a). In fact, spermidine-treated mice showed reduced visceral fat than WT controls, together with a significant decrease in the mean diameter of adipocytes (Figure 7b). Consistently, high-fat fed spermidine-treated mice showed significantly reduced hepatosteatosis when compared with untreated control mice fed with the same diet (Figure 7c). Moreover, spermidine-treated mice were significantly more glucose tolerant and insulin sensitive than untreated mice, when challenged with GTT and ITT respectively (Figures 7d and e). Several works from different labs have previously shown that spermidine treatment is able to increase autophagic flux *in vivo* in a variety of tissues, including metabolically relevant tissues as liver or skeletal muscle, but did not analyze the effect of spermidine treatment in adipose tissue.^27, 28^ As shown in Figure 7f, spermidine treatment was able to increase autophagic flux in white adipose tissue, as measured by increased LC3 lipidation accompanied by decrease in SQSTM1/p62 levels. Interestingly, the amelioration of obesity-linked parameters observed in spermidine-treated mice was associated with increased expression of ATG4B protein in spermidine-treated mice, which supports our results from *Atg4b*-null mice pointing to a protective anti-obesity role for ATG4B protease and autophagy pathway in diverse experimental settings.

Taken together, our results show that autophagic potential may influence propensity to develop different metabolic pathologies. On one hand, a partial but systemic autophagy inhibition, as in *Atg4b*-null mice^19^ or in aged individuals,^8, 29^ may predispose to obesity or diabetes upon high-calorie regimens or in circumstances that compromise pancreatic *β*-cell homeostasis. On the other hand, stimulation of autophagic flux may counteract the detrimental effects of excessive caloric intake associated with Western style nutritional habits, thus delineating new anti-obesity opportunities based on autophagy stimulation.

## Discussion

In the last years, autophagy has emerged as a pivotal pathway essential for most of cellular functions, either as a basal housekeeping process or as a stress-induced pro-survival route. Contrasting with other model organisms, such as *C. elegans* or *D. melanogaster,* in which the disruption of essential autophagy genes, such as *atg7*, is compatible with life,^30, 31^ total autophagy impairment leads to perinatal lethality in mice.^11^ To circumvent this limitation, most studies aimed to unveil the physiological functions of autophagy in mammals have relied on the analysis of mice in which autophagy was inactivated in a tissue-specific and/or a temporally-controlled fashion. These studies have revealed a pivotal role for this process in distinct metabolically relevant tissues. Specifically, autophagy impairment upon *Atg7* gene deletion in hypothalamic AgRP neurons results in reduced food intake and lean phenotype,^32^ whereas deletion of the same gene in hypothalamic pro-opiomelanocortin-producing cells leads to increased weight gain and reduced metabolic performance.^33^ Likewise, analyses of *β*-cell specific *Atg7*-deficient mice have revealed an essential role for autophagy in pancreatic islet homeostasis, in which a total loss of autophagy results in substantial cell death of *β*-cells either under standard diet or upon obesity-promoting regimens.^34^ Conversely, autophagy ablation in *Atg7*-null mice impacts adipose tissue function, leading to a lean phenotype with decreased white adipose mass and enhanced insulin sensitivity.^35, 36^ Altogether, these elegant studies have contributed to understand and dissect the role of autophagy in many different tissues, including those that are pivotal for energy homeostasis and metabolism. However, some of the results yielded by these experimental approaches may not be related to loss of tissue-specific functions of autophagy itself, but rather to tissue malfunction as a consequence of autophagy ablation, due to the housekeeping nature of basal autophagy. In fact, total impairment of of basal autophagy may compromise cellular fitness and the metabolic efficacy of affected tissues in a similar way than tissue-specific ablation of other essential processes would do, thereby masking specific and physiologically relevant functions of autophagy as an inducible anti-stress response.

**Figure 8:**
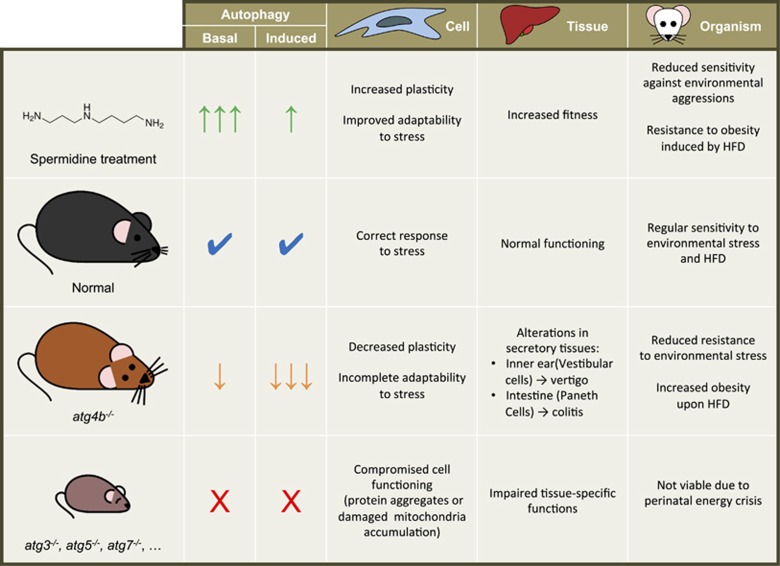
Autophagic potential influences adaptability to stress. Autophagy pathway is active in all eukaryotic cells at a basal rate, which contributes to the maintenance of cellular homeostasis. This basal autophagic activity can be further increased in response to intracellular or extracellular stimuli, including distinct forms of stress at the cell, tissue or organism level.^1^ In wild-type mice, autophagy mitigates the detrimental effects of stress, from cellular to organismal level. Contrarily, a total autophagy impairment (as it is the case in mutant mice lacking *Atg3*, *Atg5* or *Atg7*) impacts cellular homeostasis, especially in non-proliferating cells, which progressively accumulate cellular damage in the absence of autophagy.^45^ Eventually, these defects impact tissue-specific functions although the precise consequences and severity of autophagy impairment are variable among different tissues.^11^ At the organismal level, a total loss of autophagy leads to perinatal death.^2, 10^ Interestingly, a partial reduction of autophagic potential as that in *Atg4b*-deficient mice does not compromise organism viability, although leads to tissue-specific alterations^20, 46^ and reduced resilience upon stressful conditions. Finally, the pharmacological stimulation of autophagy (by the treatment with spermidine, for example) increases basal levels of the process, which improves tissue fitness and leads to a reduced sensitivity to environmental stressors, including high-fat diet

In this sense, it has to be noted that very few studies have addressed the consequences of a reduction (but not a total impairment) of autophagic activity at the whole-body level. This is a relevant question, as it is known that autophagic potential progressively declines as the organism ages^8^ and some environmental factors, including pollutants or nanoparticles, interfere with components of the pathway, reducing autophagic competency in a progressive way.^37^ The partial autophagic reduction of *atg4b*^−/−^ mice is likely more similar to real pathological conditions in which autophagic degradation is partially impaired but not totally deactivated. In fact, basal autophagic activity of *atg4b*^−/−^ mice is sufficient to achieve a lifespan almost identical to that of WT mice (our unpublished observations), with no higher incidence of any major pathology with the exception of low-penetrance early-onset balance disorder, affecting ~25% of *Atg4b*-null mice. However, *atg4b*^−/−^ cells and mice are not able to mount an appropriate autophagic response when challenged by nutrient deprivation^19^ or different autophagy-inducing agents such as the polyamine spermidine, the polyphenol resveratrol, or the mTOR inhibitor Torin (Supplementary Figure 5). Hence, the reduced but not totally impaired autophagy of *atg4b*^−/−^ mice likely affects adaptability at the cell, tissue or organism levels, thus contributing to increase susceptibility to diverse pathologies, including obesity and other metabolic disturbances (Figure 8).

ATG4B is one of the four mammalian orthologs of yeast Atg4 protease and cleaves the orthologs of Atg8 (like LC3B or GABARAP, as well as other proteins from the LC3 and GABARAP families), removing the carboxy-terminal amino acids and leaving a glycine residue at the C-terminus.^17, 38^ This cleavage is strictly required for the subsequent lipidation of the glycine residue, creating phosphatidyl ethanolamine-conjugated proteins that associate with autophagosomes.^39^ The sole Atg4 substrates identified thus far are Atg8 orthologs, suggesting that the physiological role of Atg4 is confined to autophagy. Thus, our observation that *atg4b*^−/−^ mice are prone to both experimentally induced type-I and type-II diabetes shows for the first time that reduced autophagy competency, although still sufficient to sustain tissue homeostasis in normal conditions, leads to increased sensitivity to different metabolic challenges, thus predisposing both to diet-induced obesity as well as to environmentally induced pancreatic *β*-cell loss.

Due to the pivotal importance of autophagy pathway for maintaining the homeostasis of several metabolically relevant tissues, a combination of multiple factors might underlie the observed susceptibility to obesity. For instance, *atg4b*^−/−^ mice fed upon standard chow neither show signs of increased hepatosteatosis nor liver damage. This suggests that the ability of mutant mice to perform lipophagy and other basic liver functions is sufficient to cope with specific requirements in this regard. By contrast, when subjected to pro-obesity regimens, mutant animals show clear signs of hepatosteatosis. This may partially derive from insufficient lipophagy if demands imposed by pro-obesity treatments are too high. Thus, additional systemic alterations combined with intrinsic cellular defects might likely account for the increased susceptibility of *atg4b*^−/−^ mice to experimentally induced type-I and type-II diabetes. In any case, it remains a conundrum which particular organ or organ system is more affected by autophagy deficiency in the context of obesity-linked metabolic alterations. Future studies involving the generation of tissue-specific knockout mice in which the potential to increase autophagic activity is reduced, while maintaining sufficient basal activity, as in the case of *atg4b*^−/−^ mice, may unveil these incognita. Moreover, our observation that energy expenditure is significantly reduced in autophagy-deficient mice upon pro-obesity regimens (but not under standard chow) may provide new insights in how systemic autophagic activity influences unexpected aspects of mammalian physiology.

In summary and despite of the aforementioned caveats, the present study provides strong arguments in favor of a mechanistic link between autophagy and weight control. In this context, it appears logical that prominent measures to induce autophagy (like spermidine administration or gene therapy with pro-autophagic transcription factors, such as TFEB) may inhibit or prevent obesity *in vivo*.^40, 41^

## Materials and methods

### Animals used in this study

The generation of *atg4b*^−/−^ mice has been previously described.^19^ For *atg4b*^−/−^ experiments, *Atg4b*-null mice and their corresponding wild-type (WT) littermates were derived from interbreeding of heterozygous C57Bl6/129 Sv mice and their genotypes were determined by PCR-based analysis of tail DNA. Mice were bred under specific pathogen-free conditions. All experiments were performed with 8- to 12-week-old mice and were approved by the Committee on Animal Experimentation of Universidad de Oviedo (Oviedo, Spain). For starvation-associated experiments, food was completely removed from mice cages, access to water was allowed as usual and, when indicated, mice were injected twice per day with a 300 mg/kg body weight DMKG in saline solution.

### High-fat diet treatment

Eight-week-old *atg4b*^−/−^ and their corresponding WT littermates were fed a high-fat diet containing 42% fat (Harlan TD 88137; Zeiss). Mice were weighted once a week for all the experiment. For spermidine administration, WT animals (C57BL/6 J) were intraperitoneally-injected daily with a dose of 50 mg/kg in PBS, as previously reported.^27^

### Sucrose supplementation treatment

Eight-week-old *atg4b*^−/−^ and WT mice were fed with standard rodent diet and were given a 30% sucrose solution in tap water to induce metabolic syndrome, as previously described.^42^

### Histological analysis of tissues

Tissues were harvested and further fixed with 10% neutral-buffered formalin solution (Sigma-Aldrich, St. Louis, MO, USA) and left overnight at 4 °C. After that, tissues were dehydrated, embedded in paraffin, cut and transferred into appropriate porta-slides. Slides were then deparaffinized and rehydrated. For immunohistochemistry, slides were blocked in 5% BSA (Sigma) for 10 min, incubated with primary antibodies for 1 h at 37 °C, washed in PBS, incubated for 40 min with secondary antibodies, thoroughly washed in water, and mounted. For cryosections, tissue samples were embedded in Tissue-Tek OCT compound (Sakura Finetechnical Co. Ltd., Tokyo, Japan) and stored at −80 °C. Samples were then sectioned at 5 mm thickness with cryostat (CM3050 S, Leica). Cryosections were stained with Oil Red O to detect triglycerides. For histology analysis, deparaffinized sections were stained with hematoxylin and eosin, Periodic acid–Schiff (PAS) and Masson’s Trichrome. Liver tissue sections were analyzed for inflammatory activity and steatosis. For immunofluorescence analyses, images of immunolabeled samples were obtained at room temperature with a laser-scanning confocal microscope (TCS-SP2-AOBS; Leica) Relative *β*-cell mass was estimated by point counting after insulin IHC. An average of 7000 points/mouse was counted. The number of test points was chosen to set the probable error of volume density to less than 10% of the calculated value.^43^ Histological inflammatory scores were evaluated as follows: 0, no inflammation; 1, minimal or mild focal inflammation (few neutrophils/lymphocytes); 2, multiple focal points (numerous neutrophils/lymphocytes); 3, diffuse infiltrate of neutrophils/lymphocytes of severe intensity. For the analysis of hepatic steatosis, a minimum of 100 hepatocytes per field in 5 independent fields per sample were analyzed. Those hepatocytes whose cytoplasm was mainly occupied by lipid droplets were considered steatotic hepatocytes. Pancreatic tissue sections were analyzed for necrosis of pancreatic islets as follows: 0, absence of necrosis; 1, vascular dilation; 2, mild necrosis; 3, moderate and focal or multifocal necrosis; 4, moderate and diffuse necrosis; 5, severe and focal/multifocal necrosis; 6, severe and diffuse necrosis.

### Glucose and insulin tolerance test

Prior to studies, mice were fasted overnight. For intraperitoneal glucose tolerance test (IPGTT), mice received an intraperitoneal injection of glucose (2 g/kg body weight). In intraperitoneal insulin tolerance test (IPITT) studies, mice were intraperitoneally-injected a dose of 0.75 U of insulin per kg of body weight. Blood glucose levels were determined as described above.

### Metabolic measurements

Metabolic parameters such as VO2, VCO2 and energy expenditure were obtained using the comprehensive lab animal monitoring system (Oxymax CLAMS, Columbus Instruments, Columbus, OH, USA) and analyzed following manufacturer's instruction. Mice were monitored for 48 h and the first 24 h were discarded in the analysis, considering them as acclimation period.

### Induction of diabetes

Streptozotocin (STZ; Sigma-Aldrich) was dissolved in 0.1 M sodium citrate buffer pH 4.5 immediately before its use to avoid degradation. Mice were fasted for 4 h and injected intraperitoneally the appropriate amount of the STZ solution for a final dosage of 150 mg/kg. Animals were supplied with 10% sucrose water overnight to avoid sudden hypoglycemia post injection, blood glucose was measured as described above.

### Blood and plasma parameters

Animals were fasted overnight and used for measurements of blood and plasma parameters. Blood glucose was measured with Accu-Chek glucometer (Roche Diagnostics, Barcelona, Spain) using blood from the tail vein. For all the other parameters, blood was extracted from facial vein after anesthetizing mice with halothane. Blood was immediately centrifuged at 2000 rpm in a refrigerated microfuge at 4 °C for 15 min. Supernatant (plasma) was kept at −80 °C until further analyses. For plasma insulin, leptin and adiponectin measurements, we used Millipore ELISA Kits. All protocols were performed according to manufacturer's instructions.

### Fibroblasts extraction and culture

MEFs were extracted from E13 embryos. Briefly, embryos were sterilized with ethanol, washed with PBS and triturated with razor blades. Samples were then incubated in DMEM (Gibco-Invitrogen, Carlsbad, CA, USA) overnight at 37 °C and 5% CO_2_. The next day, cultured cells were trypsinized, filtered and washed. Finally, MEFs were incubated at 37 °C and 5% CO2 and used for the corresponding experiments. For autophagy induction, MEFs were cultured during 4 h with 250 nM Torin (S2827, Selleckchem, Houston, TX, USA), 100 *μ*M spermidine (Sigma-Aldrich), 100 *μ*M Resveratrol (Sigma-Aldrich) in supplemented DMEM. For autophagy experiments, MEFs were cultured during 4 h in the presence of 100 nM Bafilomycin A1 (BML-CM110, Enzo Life Sciences, Farmingdale, NY, USA).

### Mouse experiments and tissue processing

Mice were were housed in a temperature controlled environment with 12 h light/dark cycles and received food and water *ad libitum*. Mice were injected intraperitoneally with 50 mg/kg Spd or 25 mg/kg resveratrol 6 h before anesthetization and killing. For autophagy flux experiments, mied were IP injected a 30 mg/kg single dose of Leupeptin, as previously reported.^44^ Mice tissues were immediately frozen in liquid nitrogen after extraction and homogenized in a 20 mM Tris buffer (pH 7.4) containing 150 mM NaCl, 1% Triton X-100, 10 mM EDTA and Complete protease inhibitor cocktail (Roche Applied Science). Tissue extracts were then centrifuged at 12 000 G at 4 °C and supernatants were collected. Protein concentration in the supernatants was evaluated by the bicinchoninic acid technique (BCA protein assay kit, Pierce Biotechnology, Rockford, IL, USA).

### Statistical analyses

Unless otherwise mentioned, experiments were performed in triplicate and repeated at least twice. Data were analyzed using the GraphPad Prism 5 software and statistical significance was assessed by means of two-tailed Student’s *t*-test or ANOVA tests, as appropriate (*P<0.05, **P<0.01, *n*≥3).
