# Supplementary figures and images for: Autophagy couteracts weight gain, lipotoxicity and pancreatic β-cell death upon hypercaloric pro-diabetic regimens
Source: Cell Death Dis. 2017 Aug 3;8(8):e2970–. doi: 10.1038/cddis.2017.373 (PMC5596561; doi:10.1038/cddis.2017.373)

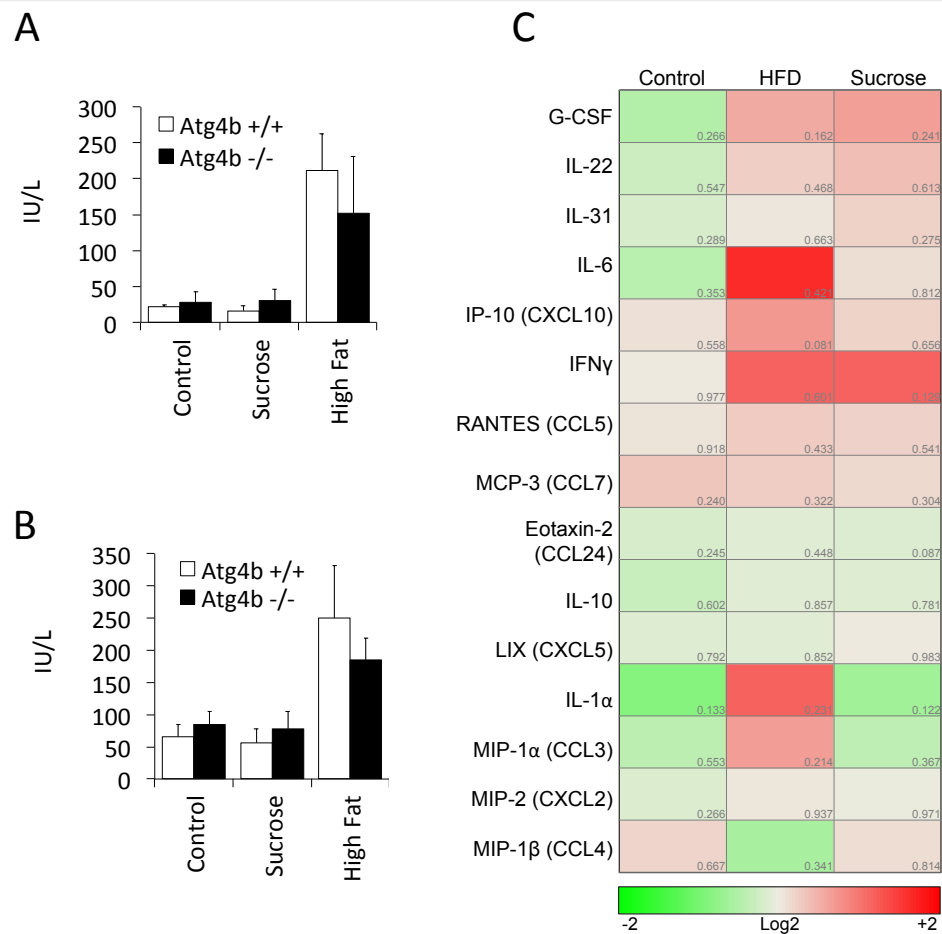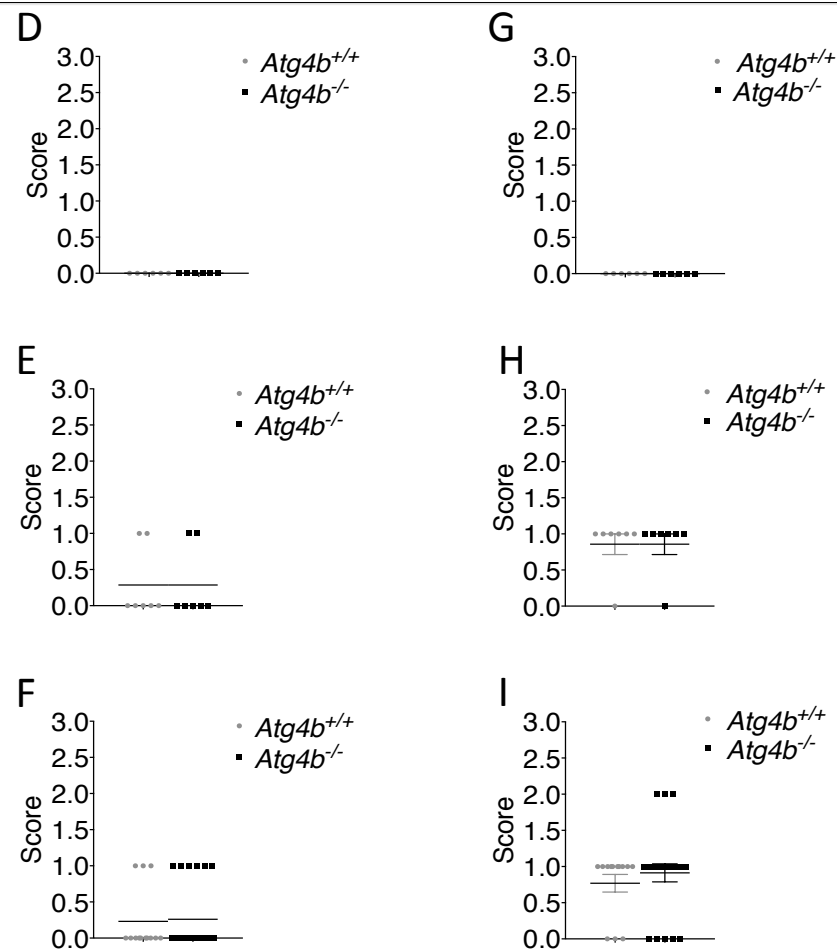

Supplemental Figure 1

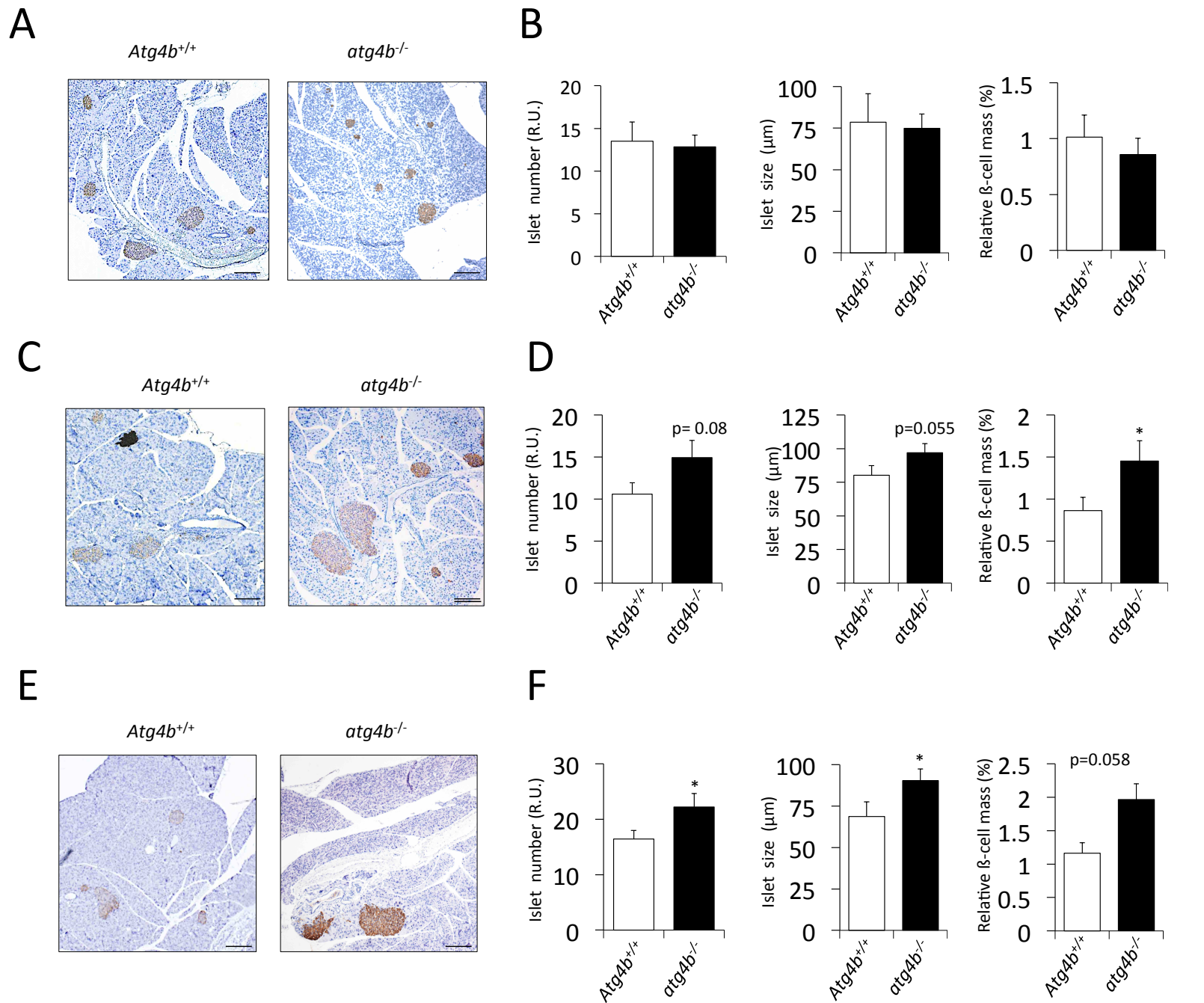

Supplemental Figure 2

**A**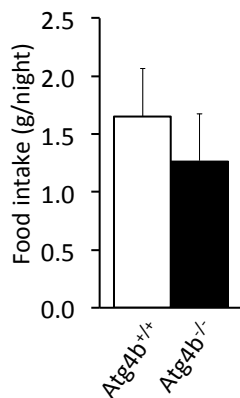**B**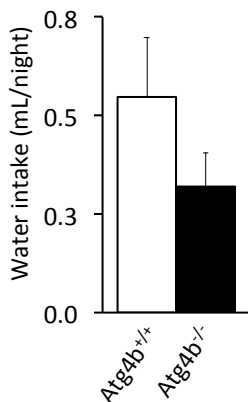**C**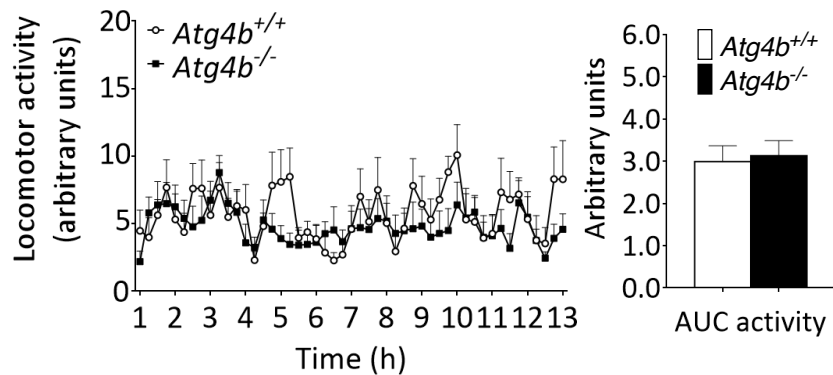**D**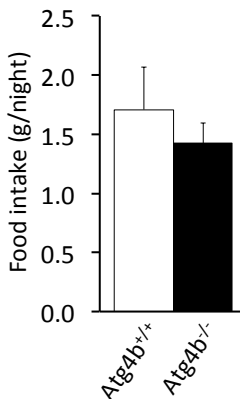**E**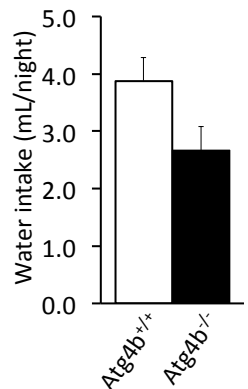**F**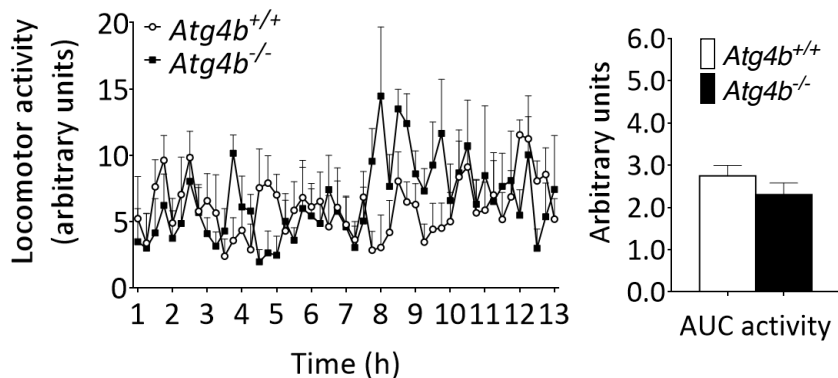**Supplemental Figure 3**

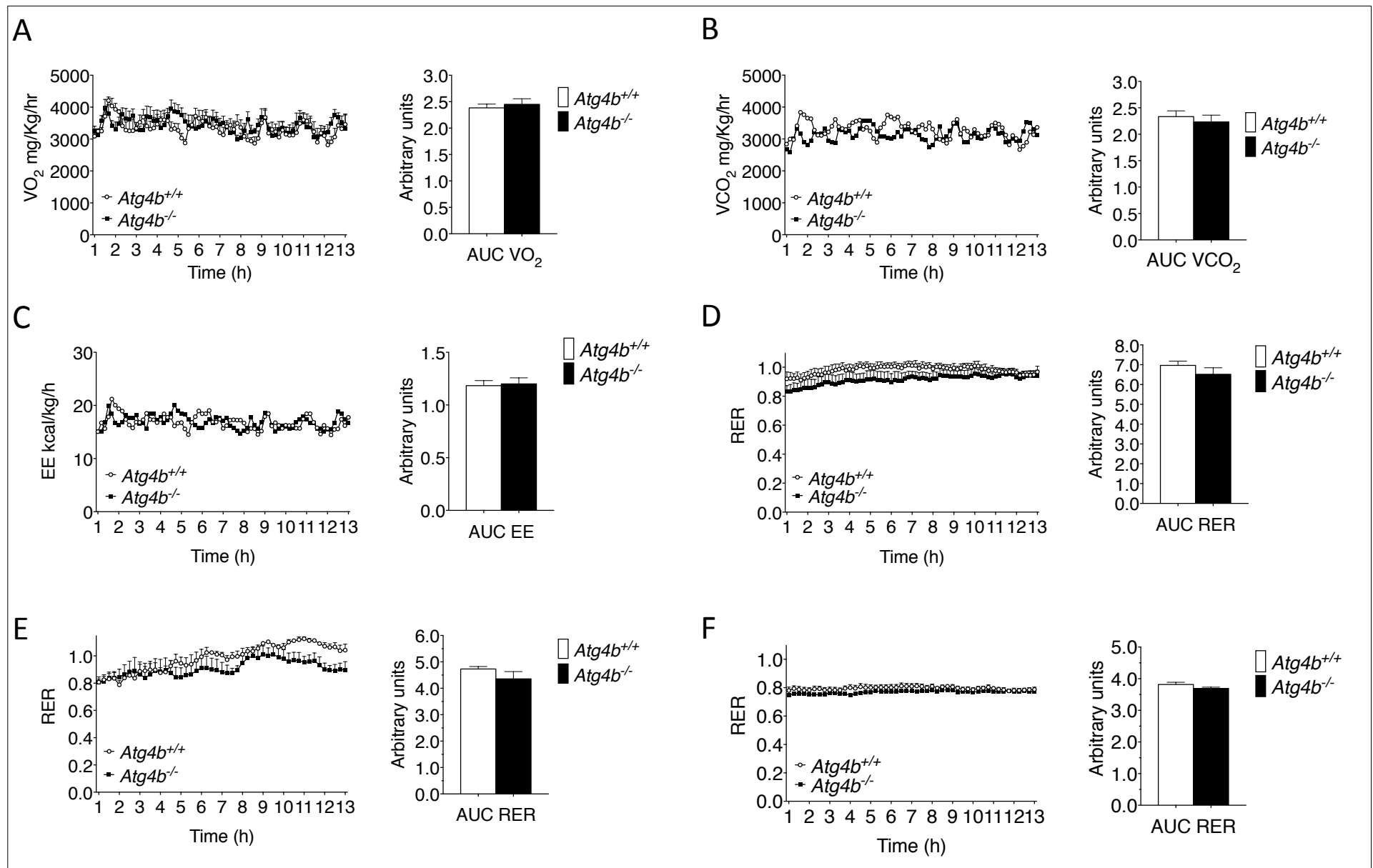

Supplemental Figure 4

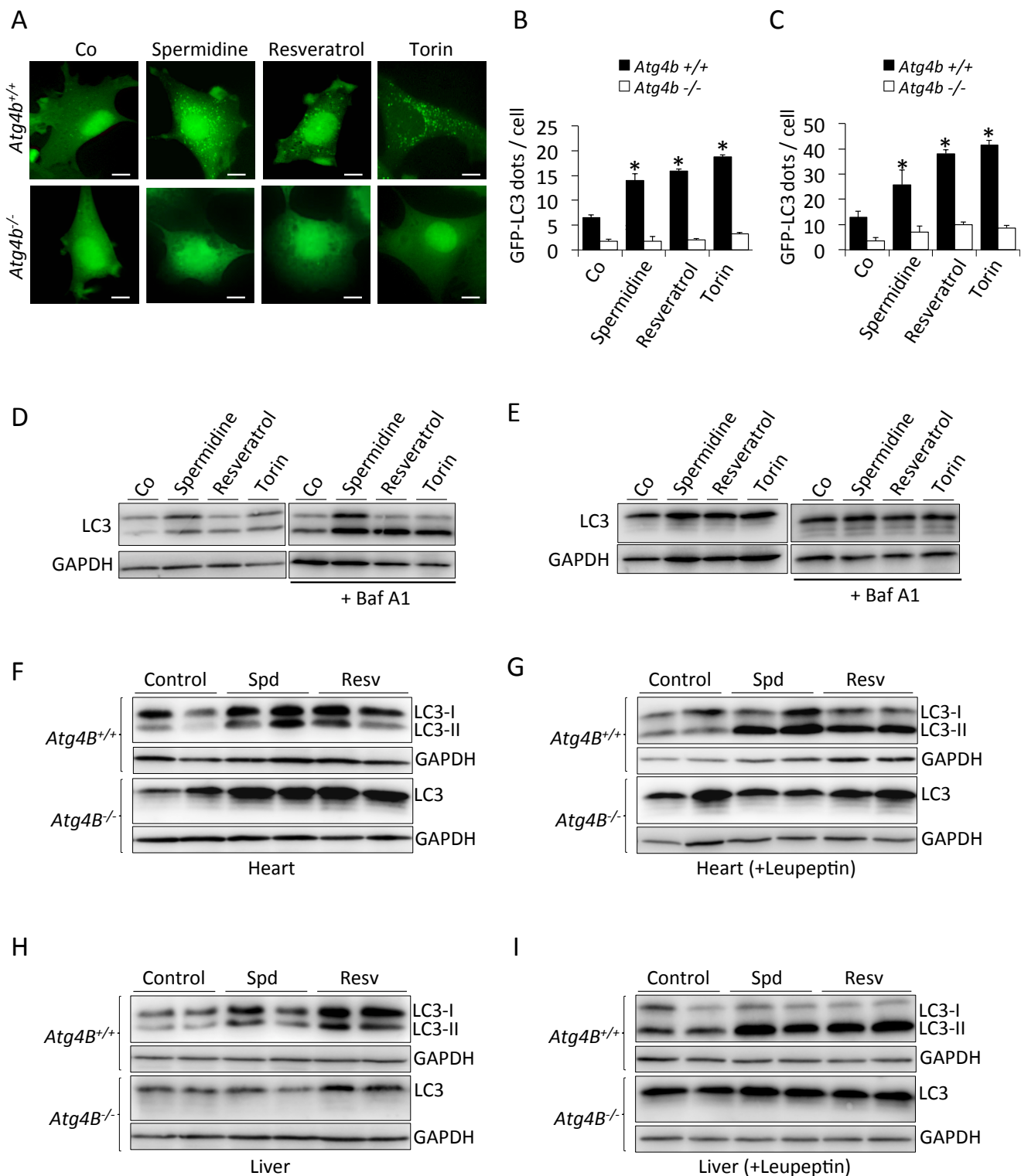

Supplement: Supplementary Figures [file cddis2017373x1.pdf]
