# Supplementary material for: Autophagy couteracts weight gain, lipotoxicity and pancreatic β-cell death upon hypercaloric pro-diabetic regimens
Source: Cell Death Dis. 2017 Aug 3;8(8):e2970–. doi: 10.1038/cddis.2017.373 (PMC5596561; doi:10.1038/cddis.2017.373)
Supplement: Supplementary Figure Legends [file cddis2017373x2.docx]

**Legends to Supplementary Figures**

**Supplemental Figure 1: A-B. AST/ALT analyses in WT or *atg4b^-/-^* mice fed upon standard chow and pro-obesity regimens. A.** AST values, as measured by International Units (IU)/ L **B.** ALT values **C-I.** **Analysis of meta-inflammation in WT or *atg4b^-/-^* mice fed upon standard chow and pro-obesity regimens.** C**.** Heat-map representing relative serum levels of pro-inflammatory cytokines and chemokines in WT and *atg4b^-/-^* mice fed upon standard chow and pro-obesity regimens. Values indicate p-values in two-tailed student t-test comparing values from WT and *atg4b^-/-^* upon the indicated treatment. **D-I.** Box-plot graphs showing inflammatory scores in adipose tissue (D-F) and liver (G-I) form WT and *atg4b^-/-^* mice fed with standard chow (D, G), high-fat diet (E, H) or fed with standard chow but supplemented with high sucrose on drinking water (F, I).

**Supplemental Figure 2: β-cell patches analyses in in *atg4b^-/-^* mice fed upon standard chow and pro-obesity regimens. A, C, E.** Representative pictures of IHC analyses against insulin, showing insulin-producing cells in pancreas sections from untreated WT and *atg4b^-/-^* mice fed with standard chow (A), high-fat diet (C) or fed with standard chow but supplemented with high sucrose on drinking water (E). **B, D, F.** Quantification of the data, showing average islet number (left graph), average islet size (middle graph) and relative β-cell mass (right graph) in pancreas from untreated WT and *atg4b^-/-^* mice fed with standard chow (B), high-fat diet (D) or fed with standard chow but supplemented with high sucrose on drinking water (F). Bars represent means ± SEM for at least 5 serial sections per mice (n>6). *, p-value <0.05 in two-tailed student’s T-test. Scale bars, 200 µm.

**Supplemental Figure 3: Analysis of food and water intake as well as locomotor activity in *atg4b^-/-^* mice fed upon high-calorie regimens**. **A-C**. CLAMS analysis of food intake (A), water intake (B) and locomotor activity (C) during dark cycle in WT and *atg4b^-/-^* mice fed with high-fat diet. **D-F.** Equivalent analyses in sucrose-treated WT and *atg4b^-/-^* mice. Data represent mean ± SEM.

**Supplemental Figure 4: Analysis of respiration and energy expenditure in *atg4b^-/-^* mice fed upon standard chow**. **A.** VO2 consumption (mL/Kg/hour) during dark phase (12 hours) (left) and area under the curve of the VO2 consumption (right) for WT and *Atg4b*-null mice upon standard diet. **B.** VCO2 production for WT and *Atg4b*-null mice fed with standard diet. **C.** Energy expenditure (Kcal/ Kg/hour) during dark phase (12 hours) and area under the curve of the energy expenditure (right) for WT and *Atg4b*-null mice fed with standard diet (n=4). **D-F.** Respiratory exchange ratio (RER) during dark phase (12 hours) (left) and area under the curve of the VO2 consumption (right) for WT and *Atg4b*-null mice upon standard diet (D), sucrose-treated (E) or fed with high-fat diet (F). Data represent mean ± SEM. *p<0.05, **p<0.01, ***p<0.001.

**Supplemental Figure 5: Autophagy flux analysis in WT and *atg4b^-/-^* MEFs and mice upon treatment with autophagy inducers. A.** Representative fluorescence pictures of WT and *atg4b^-/-^* MEF cells stably expressing the autophagosome marker GFP-LC3 after treatment with the indicated autophagy inducers for 4 h in complete culture media (Co). **B, C.** Quantification of GFP-LC3 dots for the data depicted in (A) both in the absence (B) or the presence (C) of the lysosomal inhibitor Baf A1, to measure autophagic flux. **D.** Representative immunoblots of total cell lysates for the analysis of LC3-II formation (LC3 lipidation) upon treatment with the indicated drugs both in the presence/absence of Baf A1 in WT MEFs. **E.** Equivalent analyses than in (D) in *atg4b^-/-^* cells. **F-I**. In vivo analyses of LC3 lipidation in WT or *atg4b^-/-^* mice tissue extracts upon treatment with the indicated drugs, both in the presence/absence of leupeptin (30 mg/kg) to analyze autophagic flux *in vivo*. Note the absence of LC3-II in extracts from *atg4b^-/-^* cells (E) and tissues (G, I). Blots are representative of three independent experiments yielding similar results. Graphic bars represent average and SEM values for at least 3 independent experiments. *, p-values < 0.05 in two-tailed Student’s T-test as compared to control. Scale bars, 5 µm. (n=6 for *in vivo* experiments). For cultured cells, resveratrol and spermidine were used at 100 µM and Torin was used at 250 nM. For in vivo studies, resveratrol and sperminine were intraperitonally-injected at 50 and 25 mg/kg respectively. Leupeptin was injected intraperitoneally. at 30 mg/kg. 6h after injection, mice were euthanized and organs were harvested for further processing. In leupeptin-treated groups, leupeptin injection was performed 3 hors befor e sacrifice.
